# Image Forensics in the Encrypted Domain

**DOI:** 10.3390/e26110900

**Published:** 2024-10-24

**Authors:** Yongqiang Yu, Yuliang Lu, Longlong Li, Feng Chen, Xuehu Yan

**Affiliations:** 1 College of Electronic Engineering, National University of Defense Technology, Hefei 230037, China; yy_qiang@nudt.edu.cn (Y.Y.); lilongs@nudt.edu.cn (L.L.); chenfeng23@nudt.edu.cn (F.C.); yanxh17@nudt.edu.cn (X.Y.); 2 Anhui Key Laboratory of Cyberspace Security Situation Awareness and Evaluation, Hefei 230037, China

**Keywords:** multimedia security, image forensics, copy–move detection, encrypted domain, deep learning

## Abstract

Encryption techniques used by forgers have thrown out a big possible challenge to forensics. Most traditional forensic tools will fail to detect the forged multimedia, which has been encrypted. Thus, image forensics in the encrypted domain (IFED) is significant. This paper presents the first introduction of IFED, encompassing its problem description, formal definition, and evaluation metrics. The focus then turns to the challenge of detecting copy–move alterations in the encrypted domain using the classic permutation encryption technique. To tackle this challenge, we introduce and develop a lightweight enhanced forensic network (LEFN) based on deep learning to facilitate automatic IFED. Extensive experiments and analyses were conducted to comprehensively validate the proposed scheme.

## 1. Introduction

Due to the rapid and widespread advancements in networking, mobile devices, and multimedia technology, digital images have permeated every aspect of social life. The extensive use of digital images has also driven the development and application of digital image editing software, such as Adobe Photoshop, CorelDRAW, and Meitu Xiuxiu, among others. With these editing tools, users can freely modify images to achieve improved visual effects. However, while this convenience benefits users, it also presents opportunities for illicit activities. Unauthorized individuals may engage in illegal manipulations of image content, such as non-compliant editing and the synthesis of false images, leading to the proliferation of tampered images in society. Image forensics technology was introduced in this context with the primary objective of authenticating the originality and authenticity of image data.

**Figure 1:**
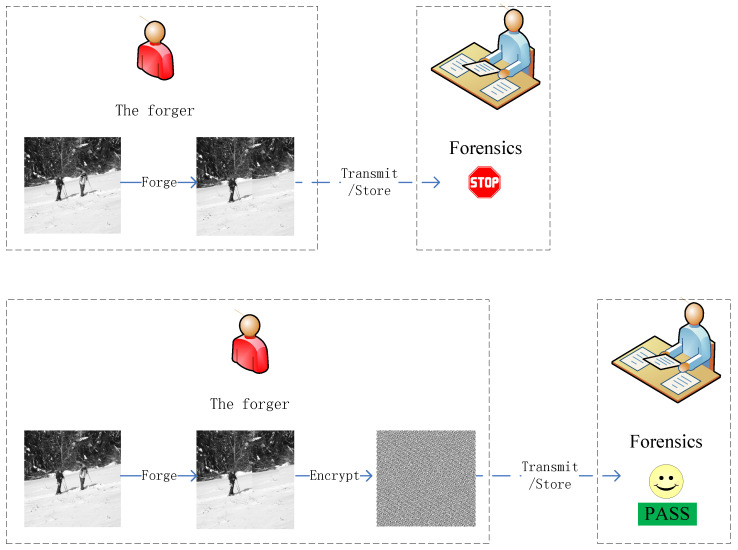
The motivation of image forensics in the encrypted domain (IFED).

Image forensics [1,2] aims to validate the origin and the authenticity of an image during transmission or in the store, as illustrated in the up part of Figure 1. Image forensics comprises two primary categories: active image forensics and passive image forensics [3,4]. Active image forensics technology refers to the proactive addition of authentication information such as signatures and watermarks [5] to images, and the detection of whether this information is damaged to authenticate the authenticity and integrity of the image. Passive image forensics is a technology that identifies the source and content authenticity of images without relying on any pre-embedded information. Passive image forensics focuses on tasks such as image source identification [6] and the detection of image forgeries [7,8,9]. Compared with active image forensics technology, passive image forensics technology has a wider range of applications and greater difficulty.

However, a forger will attack image forensics [10,11,12], namely anti-image forensics [13,14] or counter-image forensics [15]. The forger may try his best to explore the limitations of image forensics against intelligent counterfeiters, such as exploiting security weaknesses, post-processing attacks, or targeted and universal attacks to mislead forensic analyses or attack image forensic algorithms. The forger might explore using encryption technologies to mislead image forensics, as presented in the down part of Figure 1. The forger can perform encryption on the forged image, and then transmit or store the encrypted forged image to escape regulation.

Because image encryption will destroy the correlations between adjacent pixels of an image, most traditional forensic tools will fail to detect the forged image that has been encrypted. Encryption techniques used by the forger present a significant challenge to image forensics. Hence, the importance of image forensics in the encrypted domain (IFED) cannot be underestimated. As far as we know, the field of IFED is relatively uncharted and lacks prior research.

Given that IFED is fundamentally a classification problem, machine learning techniques can be employed for IFED. In recent years, deep learning has made significant advancements across various domains, showcasing its exceptional feature extraction and representation abilities, notably in computer vision and natural language processing. This paper explores the incorporation of deep learning [16] into IFED and suggests leveraging convolutional neural networks (CNNs) to develop a lightweight enhanced forensic network (LEFN). The main aim of LEFN is to tackle the challenge of automated copy–move detection within IFED.

The motivation of this paper is to introduce IFED and exploit its possibility by proposing a deep learning-based IFED. We mainly have the following contributions:

1. Image forensics in the encrypted domain is introduced, including its problem description, formal definition, and evaluation metrics.
2. A deep learning-based IFED algorithm, namely LEFN, is proposed to deal with the issue of copy–move detection, where the encryption technique of classic permutation is used. Experiments and analyses are employed to validate the proposed algorithm.
3. Compared with the traditional forensic tools that usually rely on hand-crafted features, the proposed deep learning-based LEFN can extract higher dimensional statistical features to depict the target’s potential characteristics, thus achieving possible forensics.

The structures of the following sections are as follows. Section 2 provides the foundational background for our paper. In Section 3, we present the definition of IFED. The detailed deep learning-based IFED algorithm is outlined in Section 4. Section 5 discusses the experiments and conducting comparisons. Finally, Section 6 concludes the paper by summarizing the key findings.

## 2. Preliminaries

In this section, we introduce a copy–move forgery and a traditional permutation algorithm [17] for our investigation. The essential notations used in this paper are outlined in Table 1.

### 2.1. A Copy–Move Forgery

**Figure 2:**
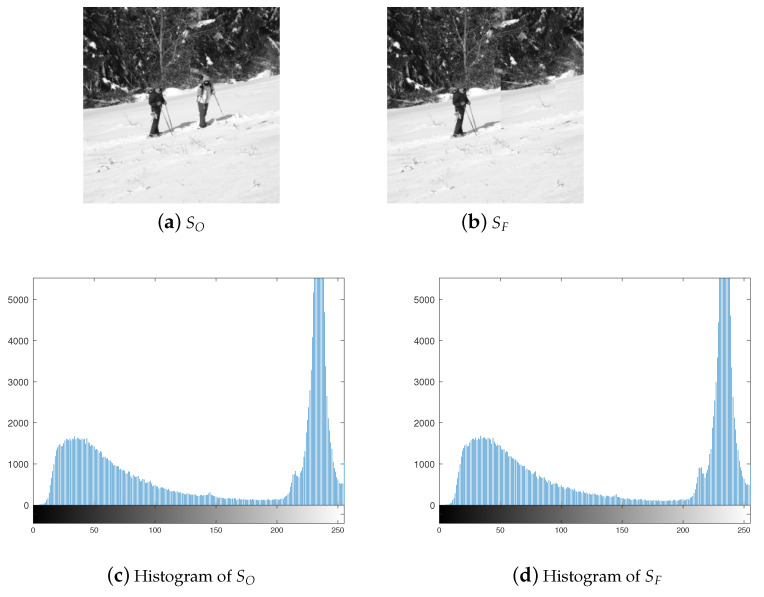
Example of one copy–move forgery.

This paper will concentrate on a particular form of forgery called copy–move, where a segment of an image is replicated and pasted within the same image. Figure 2 illustrates an instance of this type of forgery. Figure 2a depicts the original image, whereas Figure 2b presents the altered image. Figure 2c,d display the histograms of Figure 2a,b. The histograms are close to each other so that specific detection method should be designed.

### 2.2. Arnold Permutation

The Arnold permutation is a typical image permutation algorithm that can transform an original image into its encrypted form, thereby randomizing the distribution of the pixels. As shown in Equation (1), $\left(h^{\prime },w^{\prime }\right)$ denotes the position in the encrypted image corresponding to its original position (h,w) in the original image. When dealing with an image of dimensions H×W, the user can choose either *H* or *W* for the Arnold permutation. In this paper, we choose *H*. The term EI refers to the number of iterations, representing the encryption intensity.

(1) $$\left[\begin{array}{c} h^{\prime }\\ w^{\prime } \end{array}\right]={\left(\begin{array}{cc} 1 & 1\\ 1 & 2 \end{array}\right)}^{EI}\left[\begin{array}{c} h\\ w \end{array}\right]\left(\mathrm{mod}\;\;H\right)$$

**Figure 3:**
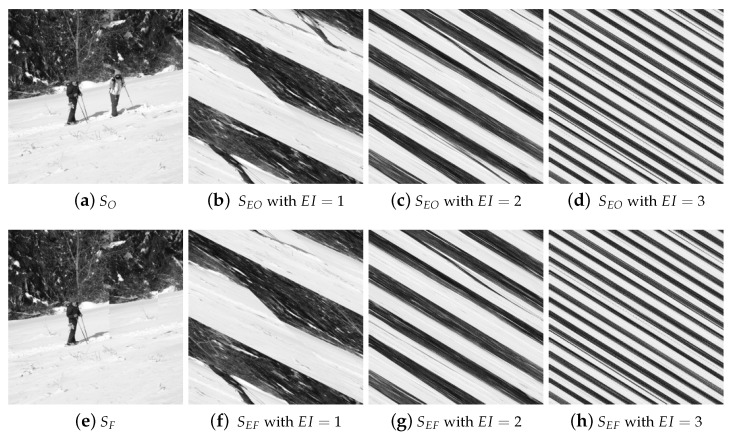
Arnold permutation illustration, where H=W=256.

Figure 3 shows the experimental results of Arnold permutation illustration, where H=W=256. The initial image is displayed in Figure 3a, and its counterfeit image is depicted in Figure 3e. Figure 3b–d display the encrypted image of Figure 3a with EI=1,2,3, respectively. Figure 3f–h demonstrate the encrypted image of Figure 3e with EI=1,2,3, respectively. According to Figure 3b–d and Figure 3f–h, we cannot discern the content of the images from their encrypted forms, demonstrating that the Arnold permutation successfully encrypts an image.

## 3. IFED Definition

**Definition** **1** (Image forensics in the encrypted domain (IFED))**.** *Image forensics in the encrypted domain (IFED) is a technique to authenticate encrypted images and establish their source and legitimacy. It is commonly categorized into active IFED and passive IFED. IFED can be categorized into the following groups based on forensic goals or tasks.*

*1*. *Source detection or identification: To detect or identify the device used to acquire the encrypted image, like a camera or scanner.*
*2*. *Forgery detection: To validate whether the encrypted image has been forged.*
*3*. *Processing operations identification: To identify the sequence of image processing.*
*4*. *Forgery cracking: To crack or recover the original image.*
*5*. *Forgery attack: To render the encrypted (forged) image unusable*

*For a forged style—and with a certain encryption algorithm and strength—we can use the following metrics to evaluate an IFED method:*

*1*. *Accuracy: The proportion of accurately classified encrypted (forged) images out of all encrypted (forged) images, represented by Equation (2).*
(2) $$Acc=\frac{TP+FN}{TP+TN+FN+FP}$$
  *where TP,TN,FN, and FP represent the quantities of true positive, true negative, false negative, and false positive classifications of encrypted (forged) images, respectively.*
*2*. *Precision: The percentage of correctly classified positive encrypted (forged) images among all encrypted (forged) images classified as positive, as shown in Equation (3).*
(3) $$Prec=\frac{TP}{TP+FP}$$
*3*. *Area under the curve (AUC) score: The possibility that a randomly selected positive encrypted (forged) image takes precedence over a randomly selected negative encrypted image.*
*Assuming there are n positive encrypted images and m negative encrypted images, AUC can be estimated through the following steps: For each positive encrypted image, compare its predicted value with the predicted values of all negative encrypted images. If the predicted value of a positive encrypted image is higher than that of a negative encrypted image, then increment the counter by one. Finally, divide the total count by the product of the number of positive encrypted images n and the number of negative encrypted images m to obtain AUC. The formulaic expression is as follows:*
(4) $$AUC=\frac{\sum_{i=1}^{n}\sum_{j=1}^{m}I\left(score\left(positive_{i}\right)>score\left(negative_{j}\right)\right)}{nm}$$
  *where I is an indicator function that returns 1 when the condition is met, otherwise it returns 0; $score(positive_{i})$ represents the predicted score of the ith positive sample; $score(negative_{j})$ represents the predicted score of the jth negative sample. The AUC value represents the area under the ROC curve, and its range is from 0 to 1. An ideal classifier should have an AUC value close to 1, while a classifier that guesses randomly would have an AUC value of about 0.5.*

We further discuss Definition 1 as follows.

1. For a given forged style, the encryption algorithm, and its EI are significant factors in evaluating an IFED method.
2. The user can select the metrics according to the practical forensic problem.

## 4. The Proposed LEFN-Based IFED Algorithm

In this section, we first introduce the design concept and general structure of the proposed LEFN before providing a comprehensive explanation of its specific modules.

### 4.1. Design Idea and Overall Architecture

As previously demonstrated and analyzed, while visually discerning between an encrypted normal image and an encrypted forged image may be challenging, their local and overall statistical characteristics are bound to differ. In particular, the tampered part will be sharply different from its adjacent regions, and it will slightly increase the histogram at certain peaks.

According to these characteristics, our designed network for IFED will contain the following several modules (design considerations):

1. A powerful feature-extraction module to extract high-level abstract features and more distinguished features between the encrypted normal image and the encrypted forged image.
2. A preprocessing module designed to better guide the feature-extraction module in focusing on sharply changing regions.
3. An enhancement module with a large receptive field to capture both local and long-range correlations, as well as the overall feature information.

For this purpose, we elaborately designed LEFN.

**Figure 4:**
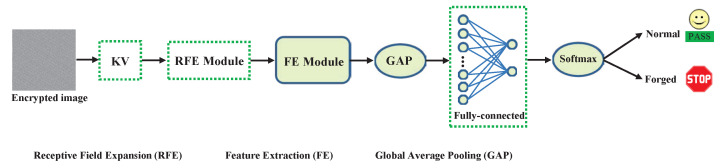
Overall architecture of the proposed LEFN.

The general structure of the suggested LEFN can be seen in Figure 4.

As depicted in Figure 4, the architecture of our developed LEFN encompasses a variety of key components, including but not limited to, the feature extraction (FE) module, global average pooling (GAP), the fully connected layers, and a softmax node. The FE module is constructed using a sequential of CNN layers to extract increasingly hierarchical abstract features to automatically model the target’s statistical distribution, eliminating the need for hand-crafted features. The global average pooling (GAP) is empirically applied to compress the extracted feature maps and it can scale the feature maps $F\in R^{H\times W}$ to $F^{\prime }\in R^{1\times 1}$, thus enabling the input image to be of any size. Furthermore, it can encode overarching data and decrease the input dimensions for the subsequent fully connected layer, leading to a notable reduction in network parameters. The ultimate classification is accomplished by the fully connected layer in conjunction with the softmax node. Alongside these fundamental modules, the KV kernel functions as a high-pass filter to differentiate the high-frequency signals that usually differentiate the variations between the encrypted authentic image and the encrypted forged image. Moreover, we also designed a receptive field expansion (RFE) module to enlarge the receptive field for capturing long-range correlations and contextual spatial information, thus hierarchically aggregating the forged information from local to global. After training and learning, without any human interference, our designed LEFN can automatically distinguish whether an encrypted image has been maliciously forged.

### 4.2. Specific Module Frameworks

In this part, we will display the specific frameworks of the above-mentioned modules and give our design considerations.

#### 4.2.1. FE Module

**Figure 5:**
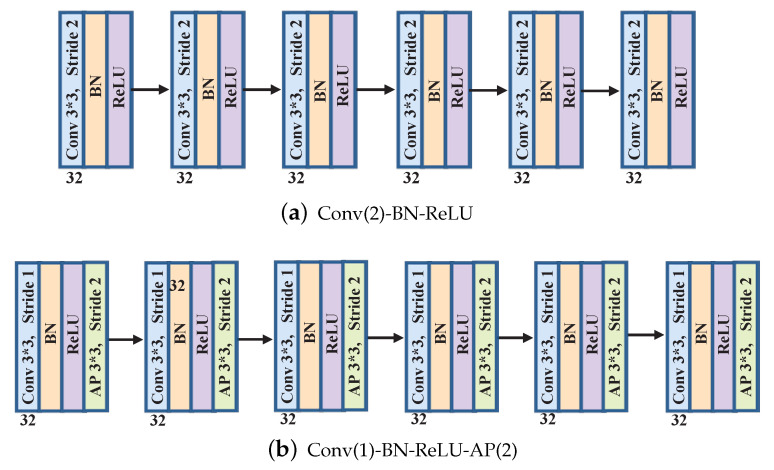
Two different frameworks of FE module (convolution (Conv), batch normalization (BN), average pooling (AP)).

The specific frameworks of the body FE module are illustrated in Figure 5.

Figure 5 displays two different frameworks of the FE module. To reduce memory consumption, we apply downsampling to compress the size of feature maps, Figure 5a,b display two classic operations for downsampling. In Figure 5a, strided convolution with a stride of 2 is directly utilized for downsampling. Following each convolutional layer, a batch normalization (BN) operation is applied to normalize all components within each feature map to possess zero mean and unit variance, thus preventing gradient back-propagation from becoming stuck in local minima. This procedure enhances the stability and speed of network training. The rectified linear units (ReLUs) function as activation functions to enhance nonlinear representation. We select 3*3 as the kernel size because it is the smallest receptive field to capture local correlations including left/center/right. Number 32 represents the channel number of each layer. Different from Figure 5a, Figure 5b applies the additional pooling layer with stride 2 to achieve downsampling, while the convolution layer is only responsible for feature extraction. Note that the above-mentioned two frameworks have the same amount of parameters.

#### 4.2.2. KV Kernel

To achieve high visual security and imperceptibility, the forger may perform slight modifications (like a kind of weak signal) to the normal image to maintain the image’s appearance unchanged. While the weak signal is essentially similar to a high-frequency noise. To guide the FE module to focus on extracting the features of the tampered location or boundary, inspired by work [18], we introduce a pre-defined KV kernel to function as a high-pass filter for isolating the high-frequency signal, which commonly indicates discrepancies between the encrypted forged image and the encrypted authentic image. The specification of the KV kernel is as follows:

(5) $$\mathrm{KV}=\frac{1}{12}\left[\begin{array}{ccccc} -1 & 2 & -2 & 2 & -1\\ 2 & -6 & 8 & -6 & 2\\ -2 & 8 & -12 & 8 & -2\\ 2 & -6 & 8 & -6 & 2\\ -1 & 2 & -2 & 2 & -1 \end{array}\right]$$

**Figure 6:**
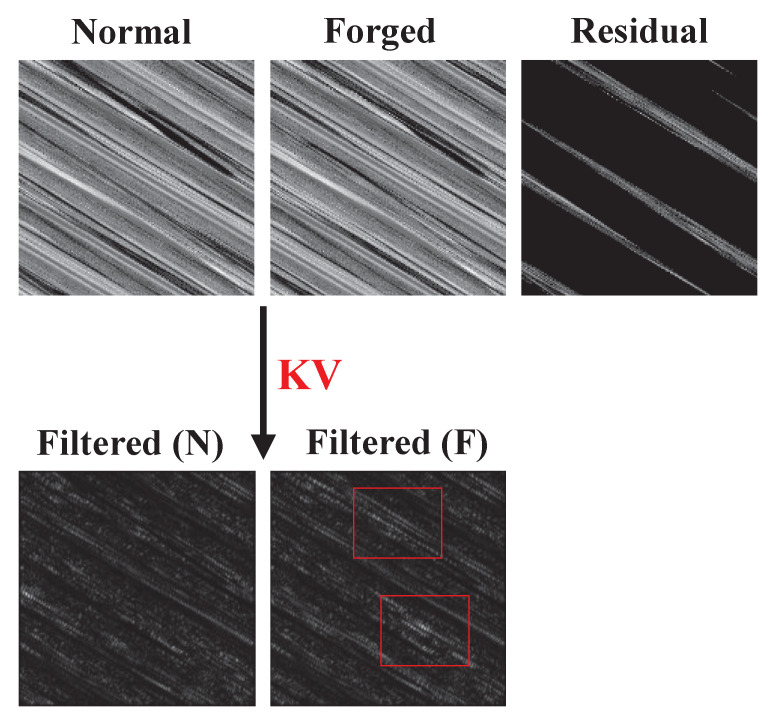
Illustration of the effect of KV kernel.

To demonstrate the impact of the KV kernel distinctly, we use a set of encrypted normal and forged images as a case paper. We showcase the resultant filtered images alongside the residual image, derived from pixel errors between the encrypted normal and forged images, in Figure 6.

In Figure 6, distinguishing any dissimilarity between the encrypted normal image and the forged image based on visual observation alone is challenging. Nevertheless, by examining the residual image formed through their pixel errors, it becomes evident how they differ distinctly. Upon applying the KV kernel to the encrypted normal image and the forged image, we derive their filtered versions. Subsequently, we identify tampered regions within the filtered forged image by highlighting them with red boxes. Obviously, the tampered regions reflected by the filtered forged images are consistent with the really tampered regions reflected by the residual image. Therefore, the KV kernel can well filter the tampered signal from the encrypted forged image and guide the next FE module to focus on extracting the feature information of the tampered signal.

#### 4.2.3. RFE Module

Although the body FE module can extract increasingly abstract features, its fixed small kernel size greatly restricts the receptive field of capturing long-range correlations. To tackle this challenge, a straightforward approach involves increasing the kernel size of convolution. However, this method leads to a significant rise in network parameters. In recent years, dilated convolution, also known as atrous convolution [19], has garnered considerable attention within the computer vision domain. This technique expands the receptive field without directly escalating the kernel size, thus circumventing the addition of parameters. It accomplishes this by inserting r−1 “holes” or zeros between consecutive kernel values, where *r* signifies the dilated rate. Describing dilated convolution using a convolution kernel W[k], the process unfolds as follows.

(6) $$O[p]=\sum_{k}I[p+r\cdot k]W[k]$$

where I corresponds to the input feature map, O pertains to the output feature map, and p denotes the location point within the feature map.

As previously mentioned, dilated convolution can expand the receptive field by introducing “holes”. However, this process leads to a gridding issue that disrupts the uniform distribution of local information. To address this issue, an effective principle [20] was proposed to eliminate the gridding effect and make the final receptive field complete without “holes”. Consider a sequential architecture consisting of *L* layers with a K×K kernel size and dilation rates of $\left[r_{1},r_{i},r_{L}\right]$. The maximum distance between two kernel values is defined as follows:

(7) $$M_{i}=\max\left[M_{i+1}-2r_{i},\;M_{i+1}-2\left(M_{i+1}-r_{i}\right),r_{i}\right]$$

and the maximum distance should satisfy the principle below:

(8) $$\left\{\begin{array}{c} M_{L}=r_{L}\\ M_{2}\leq K \end{array}\right.$$

**Figure 7:**
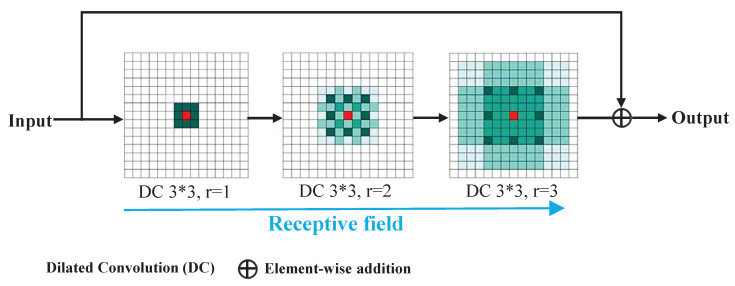
Specific frameworks of RFE module.

Following this principle, we set K=3 and utilize three consecutive convolution layers with dilation rates of r=[1,2,3] to create the Receptive Field Expansion (RFE) module, to enhance feature extraction. This approach successfully mitigates the gridding issue as $M_{2}=2<3$. Figure 7 shows the specific frameworks of the RFE module.

As depicted in Figure 7, the expansion of dilated convolution layers leads to a gradual enlargement of the receptive field for capturing surrounding information. Ultimately, the final receptive field is capable of entirely encompassing a square region. Additionally, to safeguard critical data essential for the final classification, we implement a residual connection [21] to directly transmit raw input information to the output, utilizing element-wise addition in this process.

## 5. Experimental Results and Analyses

In this section, we first set up a dataset and introduce specific experimental settings. Subsequently, we carry out thorough experiments to comprehensively showcase the efficacy of our design decisions and perform a detailed analysis on IFED.

### 5.1. Datasets and Experimental Settings

In this subsection, we briefly introduce the relevant datasets and experimental settings.

To achieve IFED, a dataset involving splicing and copy–move detection was set up as follows: We first selected 11,000 grayscale natural images, each sized 256 × 256, as the source dataset. We then encrypted the entire source dataset using Arnold permutation to obtain the encrypted normal samples. Next, we performed copy-move tampering on the entire source dataset and encrypted these tampered images to obtain the encrypted forged samples. To guarantee objectiveness and fairness, the EI was set at 2 for both normal and forged samples. From this set, 10,000 encrypted normal-forged pairs were chosen as the training set, and 1000 encrypted normal-forged pairs were selected as the testing set. It should be noted that CR (representing the level of tampering) varies from 0.25% to 25% for every 1000 images in the training set and from 0.25% to 25% for every 100 images in the testing set.

We would like to highlight that the dataset will be made available after the double-blind peer review process.

The experiments were conducted on a workstation equipped with an Intel Core i9-102920X CPU, a Titan RTX GPU with 24 GB memory, and Python 3.6 programming language using the PyTorch 1.3.0 framework on an Ubuntu 18.04 system. To optimize effectively, we used the commonly employed Adam optimizer with an initial learning rate of 0.0002 for model training. For optimization refinement, we applied the ReduceLROnPlateau tool for adaptive learning rate adjustments and set with parameters factor=0.5 and patience=5, where factor denotes the reduction proportion of the learning rate. The entire network is optimized using the cross-entropy loss function.

Since accuracy is the most important parameter in our algorithm, accuracy in Definition 1 will be adopted to evaluate the proposed IFED algorithm.

### 5.2. Ablation Study on the Network Design

In this part, we assess the efficiency of the proposed algorithm and our design choices by conducting an ablation study that involves replacing or adding specific modules.

To facilitate clarity, we will use the following abbreviations to represent the respective models, as shown in Table 2.

**Figure 8:**
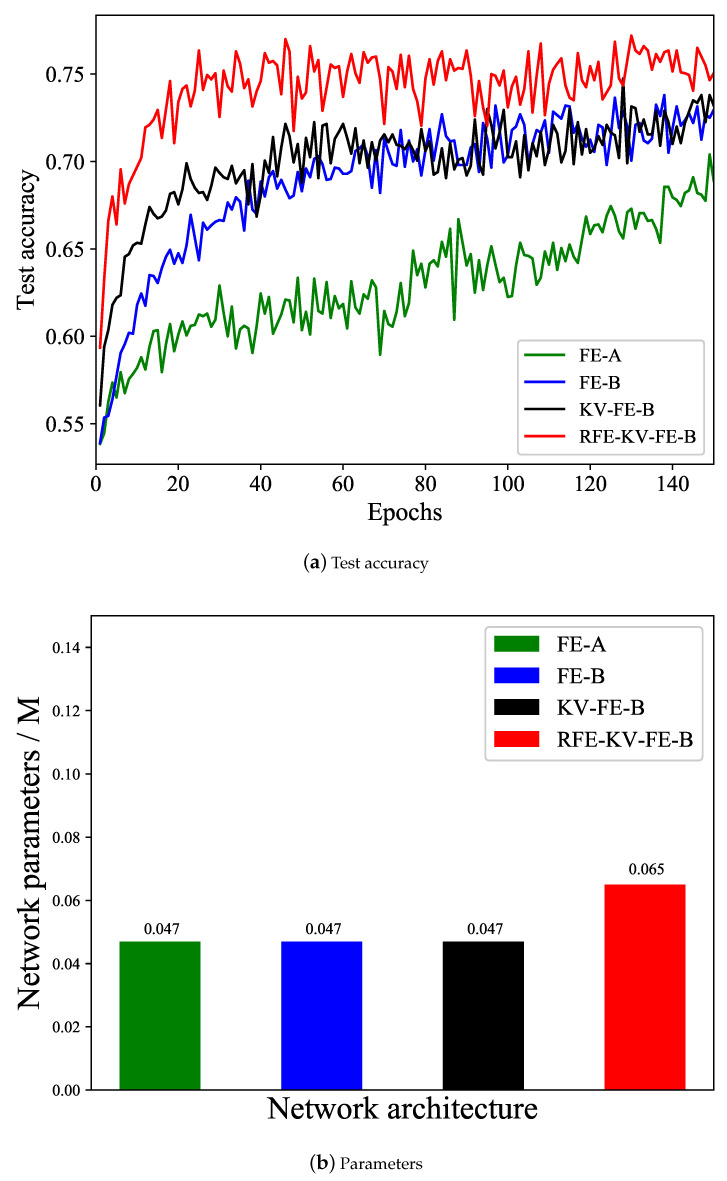
Ablation study on the network design. (**a**) Test accuracy of different network architectures; (**b**) parameters of different network architectures.

From the results displayed in Figure 8a, it is evident that as the training epochs (learning time) increase, the detection capabilities of all network models steadily enhance. And several results can be drawn from Figure 8 as follows:

1. Modules FE-A and FE-B highlight the crucial function of the average pooling operation in LEFN. This operation retains the comprehensive feature information and captures statistical characteristics by progressively condensing the feature maps. Furthermore, the additional average pooling operation behind the convolution layer will further provide stronger constraints for the local or overall correlations of the extracted feature maps, thus greatly improving the detection accuracy.
2. Modules FE-B and KV-FE-B verify the effectiveness of the KV kernel, it works as a high-pass filter to screen the high-frequency residual signal in the tampered region and conduct the next FE module to locate the important clues for feature extraction, thus speeding up network training and learning.
3. Modules KV-FE-B and RFE-KV-FE-B showcase the vital importance of the RFE module within LEFN. This module proficiently expands the receptive field to encompass distant surrounding information and global semantic details, thereby markedly enhancing the model’s learning efficiency and detection accuracy.
4. Figure 8b indicates that our designed full model is lightweight with only 0.065 M parameters, and the designed modules significantly enhance the network performance without dramatically increasing the model parameters.

### 5.3. Influence of CR on IFED

To further demonstrate the influence of CR on IFED, we show the average detection accuracy on the testing set with CR ranging from 0.25% to 25% in Table 3.

Based on the data provided in Table 3, the following conclusions can be drawn.

1. Generally, higher CR means higher detection accuracy. When CR is lower than 9%, the encrypted forged image is less likely to be detected, when CR is higher than 9%, so the encrypted forged image is more likely to be detected. Because higher CR means larger modifications and more distinguishable features.
2. Due to the integrated modules, the comprehensive RFE-KV-FE-B model attains the highest detection performance, aligning with the findings depicted in Figure 8a.

### 5.4. Influence of EI on IFED

**Figure 9:**
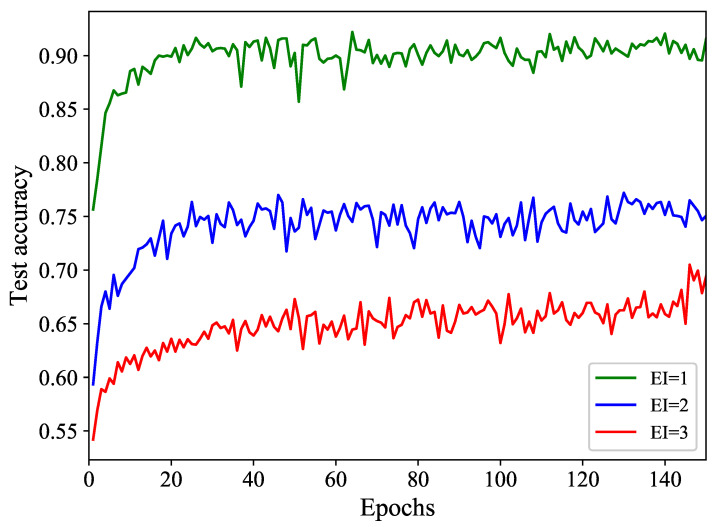
Detection accuracy corresponding to different EIs.

To demonstrate the influence of EI on IFED, we re-encrypt the above-mentioned datasets with a lower EI of 1 and a higher EI of 3. To ensure a fair comparison, the CR remains consistent as previously stated. The outcomes are depicted in Figure 9.

In Figure 9, our model’s detection accuracy decreases as EI rises, aligning with practical scenarios. This can be explained by noting that a higher Encryption Intensity (EI) means increased encryption complexity, thus dramatically boosting the detection difficulty.

### 5.5. Deal with the Increase of EI

**Figure 10:**
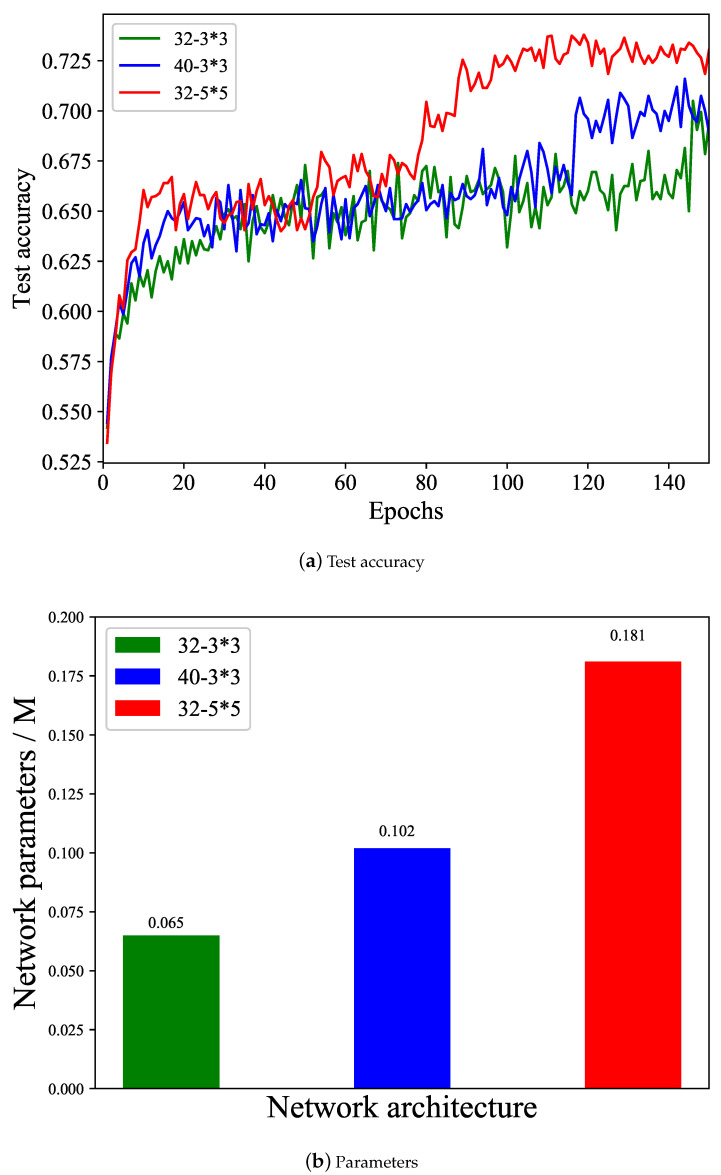
Validation of adding network parameters. (**a**) Test accuracy of different network architectures; (**b**) parameters of different network architectures.

Here arises an intuitive question: Is there any way to deal with the increase in EI? Considering that the encryption complexity has increased in practice, the model for detection will also need to become more robust. To address this, we explore options such as increasing the parameters of our model by expanding the feature channels or enlarging the kernel size. To ensure a fair comparison, EI is set at 3, and the results are displayed in Figure 10.

As shown in Figure 10, 32-3*3 represents the designed initial model with 32 feature channels and a 3*3 kernel size; 40-3*3 represents the network model with 40 feature channels and a 3*3 kernel size; and 32-5*5 represents the network model with 32 feature channels and a 5*5 kernel size.

From Figure 10, we can obtain the following results:

1. Increasing the feature channels will increase the network parameters and the detection accuracy is accordingly improved.
2. Enlarging the kernel size will dramatically increase the network parameters; however, it also significantly boosts the detection accuracy.

The above results demonstrate that a larger network is usually required when EI is higher, which verifies the effectiveness of adding network parameters to deal with the increase in EI. And the users can flexibly adjust the network structure according to the actual platform resources.

### 5.6. Discussions

We only begin the first step. According to the dataset and the proposed IFED algorithm, only a simple copy–move operation and classic permutation are considered. The proposed IFED algorithm (LEFN) may not work when more manipulated operations and complex encrypted techniques are utilized.

In the future, we can further extend our work in the following ways.

1. More digital image processing operations can be tested, such as splicing, rotating, and compressing. Their corresponding datasets can be set up.
2. Some other encrypted techniques will be exploited and used, such as Paillier cryptosystem-based proxy encryption, homomorphic wavelet transform, and Lattice-based homomorphic cryptosystems.
3. Applying digital watermarking in the encrypted domain to IFED enables the realization of active IFED, as demonstrated by [22,23,24].
4. Since image encryption might not modify the EXIF (exchangeable image file format) information, source detection or identification of IFED will be possible.
5. A specific deep learning network for IFED will be designed.
6. We can design more forensic methods for IFED. One potential approach is as follows: IFED is analogous to a ciphertext-only attack, where some plaintext is replaced within a given encryption algorithm. Therefore, we may achieve IFED from the perspective of cryptanalysis.
7. Real-time IFED based on traffic is significant.

## 6. Conclusions

This paper introduces a precise definition of image forensics in the encrypted domain (IFED), encompassing its problem description, formal definition, and evaluation metrics. Aiming at a typical copy–move detection issue within IFED, this paper employs deep learning to design a network named LEFN, which is data-driven and can automatically achieve IFED without any human intervention. Comprehensive experiments were conducted to confirm the efficacy of the developed modules. This work is exploratory research on the issue of image forgery encryption detection (IFED) and many problems remain. For example, increasing the encryption intensity dramatically deteriorates model performance. Nevertheless, this work establishes a solid baseline and paves the way for further research on IFED.

## Figures and Tables

**Table 1:** Key notations.

Notation	Description
H×W	The size of the image
$S_{O}$	The original (normal) image
$S_{EO}$	The encrypted original image
$S_{F}$	The forged image
$S_{EF}$	The encrypted forged image
EI	The encryption intensity
CR	The copy–move forgery rate
IFED	Image forensics in the encrypted domain
LEFN	Lightweight enhanced forensic network

**Table 2:** The model used in the ablation study.

Model Name	Model Description
FE-A	Model featuring only the FE module from Figure 5a
FE-B	Model containing solely the FE module shown in Figure 5b
KV-FE-B	Model incorporating the KV kernel and FE module depicted in Figure 5b
RFE-KV-FE-B	Model equipped with the RFE module, KV kernel, and FE module as shown in Figure 5b

**Table 3:** Average detection accuracy on the testing set with different CRs.

CR (%)	0.25	1	2.25	4	6.25	9	12.25	16	20.25	25
FE-A	62%	65%	70%	74%	68%	75%	75%	73%	71%	73%
FE-B	63%	71%	67%	74%	71%	83%	75%	81%	77%	79%
KV-FE-B	66%	70%	67%	79%	72%	80%	76%	82%	79%	79%
**RFE-KV-FE-B**	**67%**	**72%**	**70%**	**77%**	**77%**	**83%**	**81%**	**83%**	**80%**	**86%**

## Data Availability

The original contributions presented in the study are included in the article, further inquiries can be directed to the corresponding author.
